# Defining an Optimal Cut-Point Value in ROC Analysis: An Alternative Approach

**DOI:** 10.1155/2017/3762651

**Published:** 2017-05-31

**Authors:** Ilker Unal

**Affiliations:** School of Medicine, Department of Biostatistics, Cukurova University, Saricam, Adana, Turkey

## Abstract

ROC curve analysis is often applied to measure the diagnostic accuracy of a biomarker. The analysis results in two gains: diagnostic accuracy of the biomarker and the optimal cut-point value. There are many methods proposed in the literature to obtain the optimal cut-point value. In this study, a new approach, alternative to these methods, is proposed. The proposed approach is based on the value of the area under the ROC curve. This method defines the optimal cut-point value as the value whose sensitivity and specificity are the closest to the value of the area under the ROC curve and the absolute value of the difference between the sensitivity and specificity values is minimum. This approach is very practical. In this study, the results of the proposed method are compared with those of the standard approaches, by using simulated data with different distribution and homogeneity conditions as well as a real data. According to the simulation results, the use of the proposed method is advised for finding the true cut-point.

## 1. Introduction

The ROC curve is a mapping of the sensitivity versus 1 − specificity for all possible values of the cut-point between cases and controls. To measure the diagnostic ability of a biomarker, it is common to use summary measures such as the area under the ROC curve (AUC) and/or the partial area under the ROC curve (pAUC) [1]. A biomarker with AUC = 1 discriminates individuals perfectly as diseased or healthy. Meanwhile, an AUC = 0.5 means that there is no apparent distributional difference between the biomarker values of the two groups [2].

ROC analysis provides two main outcomes: the diagnostic accuracy of the test and the optimal cut-point value for the test. Cut-points dichotomize the test values, so this provides the diagnosis (diseased or not). The identification of the cut-point value requires a simultaneous assessment of sensitivity and specificity [3]. A cut-point will be referred to as optimal when the point classifies most of the individuals correctly [4, 5].

AUC, sensitivity, and specificity values are useful for the evaluation of a marker; however they do not specify “optimal” cut-points directly. In the literature, related to the subject, there are many approaches using both sensitivity and specificity for cut-point selection [4–9]. One of the commonly used method is the Youden index (*J*) method [5]. This method defines the optimal cut-point as the point maximizing the Youden function which is the difference between true positive rate and false positive rate over all possible cut-point values [6, 7]. Another approach is known as the point closest-to-(0,1) corner in the ROC plane (ER) which defines the optimal cut-point as the point minimizing the Euclidean distance between the ROC curve and the (0,1) point [4]. A third approach is based on the maximum achievable value of the chi-square statistic (min⁡*P*) which is driven using the cross-tabulations of true disease status and categorized new variables that separate the biomarker into two categories according to all possible cut-point values [8]. A more recent approach was proposed by Liu [9], which defines the optimal cut-point as the point maximizing the product of sensitivity and specificity (CZ). In the literature, there are studies comparing optimal metrics derived from the sensitivity, specificity, agreement, and distance [10, 11]. In these studies, it is generally recommended that researchers should select one that is most clinically relevant.

In this study, a new approach is proposed for the identification of the optimal cut-point value in ROC analysis. The approach is based on the area under the ROC curve (AUC), sensitivity, and specificity values. It defines the optimal cut-point value as the point minimizing the summation of absolute values of the differences between AUC and sensitivity and AUC and specificity provided that the difference between sensitivity and specificity is minimum.

In the following section, first the background methodologies of previous methods are summarized, and, then, the proposed method is introduced. In Section 3, in order to compare the performance of the previous methods with that of the proposed one, generated data under the assumption of normal distribution and gamma distribution models for the biomarker are used. Then, in Section 4, using data from a real-world study of heart-failure patients [12], the cut-points for pulse pressure, plasma sodium, LVEF, and heart rate in prediction of mortality are calculated by applying the proposed and the previous methods. Finally, in Section 5, conclusions are given.

## 2. Previous Methods and the Proposed Method

### 2.1. Minimum *P* Value Approach (min⁡*P*)

Let *X* be a continuous biomarker that is assumed to be predictive of an event *E* (i.e., *E* = 1 for diseased or *E* = 0 for not diseased). At any given possible cut-point *c* of *X*, sensitivity (Se) and specificity (Sp) values are as follows:

(1) Sec=PX>c ∣ E=1,Spc=PX≤c ∣ E=0.

Cut-point *c* separates the data into two groups which forms a 2 × 2 table, as shown in Table 1.

The minimum *P* value approach was proposed by Miller and Siegmund [8] and defines the optimal point as cut-point ${\hat{c}}_{\min P}$ that maximizes the standard chi-square statistic with one degree of freedom:

(2) $$\begin{array}{c} \chi_{1}^{2}\left(\;c\;\right)=\frac{N{\left(sv-ur\right)}^{2}}{\left(s+r\right)\left(u+v\right)\left(s+u\right)\left(r+v\right)}, \end{array}$$

where *N* = *s* + *r* + *u* + *v*. As it was shown by Rota and Antolini [11], it can be also written in terms of classification probabilities:

(3) $$\begin{array}{c} \chi_{1}^{2}\left(\;c\;\right)=\frac{{\left(Se\left(c\right)+Sp\left(c\right)-1\right)}^{2}}{\left(\left(\left(u+v\right)Se\left(c\right)+\left(s+r\right)\left(1-Sp\left(c\right)\right)\right)/N\right)\left(1-\left(\left(u+v\right)Se\left(c\right)+\left(s+r\right)\left(1-Sp\left(c\right)\right)\right)/N\right)\left(1/\left(u+v\right)+1/\left(s+r\right)\right)}. \end{array}$$

### 2.2. Youden Index (*J*)

The Youden index (*J*) is a measure for evaluating the biomarker effectiveness. This measure was first introduced to the medical literature by Youden [5]. *J* is a function of Se(*c*) and Sp(*c*), such that

(4) $$\begin{array}{c} J\left(\;c\;\right)=\left\{\;Se\left(\;c\;\right)+Sp\left(\;c\;\right)-1\;\right\}=\left\{\;Se\left(\;c\;\right)-\left(\;1-Sp\left(\;c\;\right)\;\right)\;\right\} \end{array}$$

over all cut-points *c*; ${\hat{c}}_{J}$ denotes the cut-point corresponding to *J*. When the value of *J* is maximum, ${\hat{c}}_{J}$ is the “optimal” cut-point value [6, 7].

### 2.3. The Closest to (0,1) Criteria (ER)

In this criteria, the “optimal” cut-point is defined as the point closest to the point (0,1) on the ROC curve [3, 4].

(5) $$\begin{array}{c} ER\left(\;c\;\right)=\left(\;\sqrt{{\left(1-Se\left(c\right)\right)}^{2}+{\left(1-Sp\left(c\right)\right)}^{2}}\;\right). \end{array}$$

Mathematically, the point ${\hat{c}}_{ER}$ minimizing the ER(*c*) function is called the “optimal” cut-point value.

### 2.4. Concordance Probability Method (CZ)

The concordance probability method proposed by Liu [9] defines the optimal cut-point as the point maximizing the product of sensitivity and specificity.

(6) $$\begin{array}{c} CZ\left(\;c\;\right)=Se\left(\;c\;\right)*Sp\left(\;c\;\right). \end{array}$$

This product gets value between 0 and 1. The concordance probability of dichotomized measure at cut-point *c* can be expressed as the area of a rectangle associated with the ROC curve. Cut-point ${\hat{c}}_{CZ}$ maximizing CZ(*c*) actually maximizes the area of the rectangle [9].

### 2.5. The Proposed Method: Index of Union (IU)

Perkins and Schisterman [4] stated that the “optimal” cut-point should be chosen as the point which classifies most of the individuals correctly and thus least of them incorrectly. From this point of view, in this study, the Index of Union method is proposed. This method provides an “optimal” cut-point which has maximum sensitivity and specificity values at the same time. In order to find the highest sensitivity and specificity values at the same time, the AUC value is taken as the starting value of them. For example, let AUC value be 0.8. The next step is to look for a cut-point from the coordinates of ROC whose sensitivity and specificity values are simultaneously so close or equal to 0.8. This cut-point is then defined as the “optimal” cut-point. The above criteria correspond to the following equation:

(7) $$\begin{array}{c} IU\left(\;c\;\right)=\left(\;\left|\;Se\left(\;c\;\right)-AUC\;\right|+\left|\;Sp\left(\;c\;\right)-AUC\;\right|\;\right). \end{array}$$

The cut-point ${\hat{c}}_{IU}$, which minimizes the IU(*c*) function and the |Se(*c*) − Sp(*c*)| difference, will be the “optimal” cut-point value.

In other words, the cut-point ${\hat{c}}_{IU}$ defined by the IU method should satisfy two conditions: (1) sensitivity and specificity obtained at this cut-point should be simultaneously close to the AUC value; (2) the difference between sensitivity and specificity obtained at this cut-point should be minimum. The second condition is not compulsory, but it is an essential condition when multiple cut-points satisfy the equation.

In order to illustrate how the IU method defines the “optimal” cut-point, the values obtained from an artificial data are used. Some of the cut-points (with their sensitivity and specificity values) provided by the artificial data are given in Table 2. In this example, the AUC value is calculated as 0.918. For the sake of simplicity, instead of 1 − specificity values, specificity values are given in the table. By using IU method, one can easily find that sensitivity (0.92) and specificity (0.92) values of the cut-point 1.985 are the nearest ones to the AUC value. Since also the difference between these two values is minimum, this cut-point will be called the “optimal” cut-point by the IU method.

However, it should be noted that choosing such a cut-point as the “optimal” cut-point may sometimes fail. For example, let Se(*c*) = Sp(*c*) = AUC = 0.8. Then, the IU(*c*) statistic given in (7) will be 0 and also the difference between Se(*c*) and Sp(*c*) will be 0. Thus according to the definition of optimality given in the IU method, cut-point *c* will be accepted as the “optimal” cut-point. However, if there is a point *c*^*∗*^ for which Se(*c*^*∗*^) = 0.82 and Sp(*c*^*∗*^) = 0.80, then the total misclassification rate will be 0.38 (which is smaller than that of the point *c*, i.e., 0.40). Hence, cut-point *c*^*∗*^ is a better optimized point than cut-point *c*, based on the definition of optimality given by Perkins and Schisterman [4].

**Figure 1:**
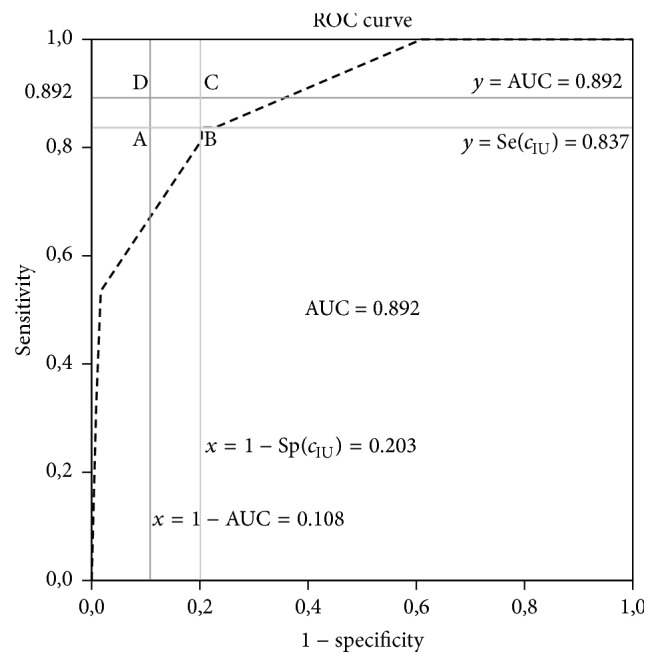
The receiver operator characteristic curve for pulse pressure in the prediction of cardiovascular death [12].

Geometrically, the idea behind the IU method is very similar to the idea behind the ER method. As it can be seen in Figure 1, the IU method also tries to find the closest point to a point, that is, the point (1 − AUC, AUC). In the ER method, this point is taken as (0,1). However, instead of using the Euclidean distance as in the ER method, the IU method uses the absolute differences between the diagnostic accuracy measures and the AUC value. More specifically, the IU method searches for the point that minimizes the half perimeter of the ABCD rectangle seen in Figure 1. This rectangle is constructed by connecting the intersections points of the lines of *x* = 1 − AUC, *y* = AUC, *x* = 1 − Sp(*c*), and *y* = Se(*c*).

## 3. Simulation Study

As it was shown by Rota and Antolini [11] although some of these methods are mathematically related, they do not necessarily identify the same true cut-point. That is, depending on the design of the study (balanced or unbalanced), the methods may identify different cut-points. According to their results, in the balanced homoscedastic scenario, the methods identified the same point; in the remaining scenarios (i.e., unbalanced homoscedastic and balanced/unbalanced heteroscedastic scenarios), the methods identified different cut-points. These results emphasize the importance of correctly defining the true cut-point in all possible scenarios.

Let us assume that a specific biomarker (*X*) in diseased and nondiseased populations is normally distributed, *X*_1_ ~ *N*  (*μ*_1_, *σ*_1_ = 1) for diseased subjects and *X*_0_ ~ *N*  (0, *σ*_0_ = 1) for nondiseased subjects. Under these assumptions, sensitivity and specificity can be written as

(8) Sec=PX1≥c=Φμ1−c,Spc=PX0≤c=Φc,

where Φ denotes the standard normal distribution function. The optimal cut-point occurs at the intersection of the normal probability density functions of diseased and nondiseased subjects (i.e., *c*_opt_ = *μ*_1_/2) [7, 13]. For example, if *μ*_1_ is taken as {0.51,1.05,1.68,2.56}, the corresponding true cut-points will be *c*_opt_ = {0.25,0.52,0.84,1.28} [11, 13]. These values of *μ*_1_ guarantee a wide variety of classification accuracies, ranging from a poor to a high one [7, 11, 13]. The identification of the true theoretical cut-point for the IU method under this scenario is given in the Appendix.

**Figure 2:**
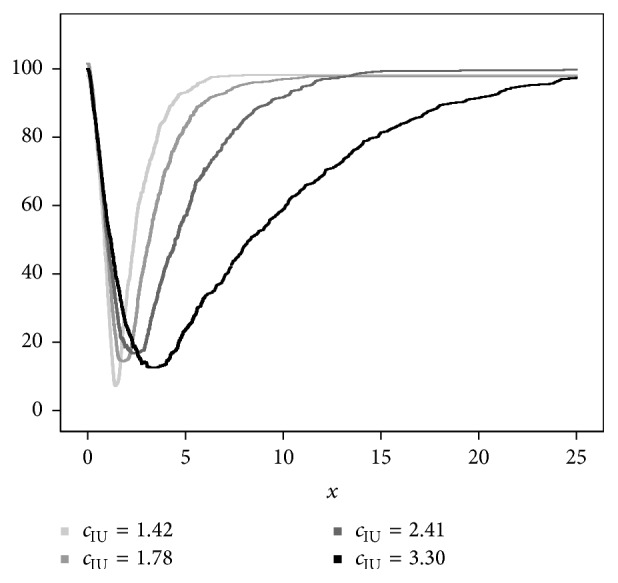
The empirically estimated objective functions IU(*c*) under different underlying distributions: light to dark colors represent the scenarios with the classification accuracies from poor to high one. The homoscedastic gamma distribution scenario with a balanced design (*n*_0_ = *n*_1_ = 100) is represented.

Now assume that *X* is gamma distributed with the following parameters: *X*_1_ ~ *G*  (*α*_1_ = 2.5, *β*_1_) for diseased subjects and *X*_0_ ~ *G*  (*α*_0_ = 1.5, *β*_0_ = 1) for nondiseased subjects. If, for instance, *β*_1_ is taken as {0.79, 1.22, 1.97, 3.82}, the corresponding cut-points for each method will be different; that is, for min⁡*P* approach, *c*_min⁡*P*_ = {0.80,1.73,2.54,3.51}, for Youden index, *c*_*J*_ = {1.12,1.79,2.45,3.42}, for the concordance probability, *c*_CZ_ = {1.35,1.81,2.41,3.38}, and, for the point closest-to-(0,1) corner, *c*_ER_ = {1.38,1.82,2.36,3.24} [11]. For the Index of Union, the corresponding cut-points are estimated by the empirical estimation method given in Liu's work [9] as *c*_IU_ = {1.42,1.78,2.41,3.30} (Figure 2).

In order to compare the performance of the cut-point selection methods with the performance of the method proposed in this study, a simulation study is conducted with different scenarios. These scenarios are the same as the ones given in Rota and Antolini's work [11]. The first scenario is normal homoscedastic scenario with balanced design where all of the methods theoretically identify the same true cut-point. The second one is the nonbalanced normal case, where all of the methods except the min⁡*P* approach identify the same cut-point. The last scenario is gamma case where all of the methods identify different cut-points.

In all scenarios, 1000 samples were generated with sample sizes 50, 100, and 200 for each group and with sample size *n*_1_ = 50, *n*_0_ = 100; *n*_1_ = 50, *n*_0_ = 150; and *n*_1_ = 50, *n*_0_ = 200 (*n*_1_ is the number of diseased subjects and *n*_0_ is the number of nondiseased subjects).

For each sample, the optimal cut-points ${\hat{c}}_{\min P}$, ${\hat{c}}_{J}$, ${\hat{c}}_{CZ}$, ${\hat{c}}_{ER}$, and ${\hat{c}}_{IU}$ for the minimum *P* value, the Youden index, the concordance probability, the point closest-to-(0, 1) corner, and the Index of Union are estimated, respectively. The relative bias and mean square error (MSE) values of each method are computed by $E\left[\left(\hat{c}-c\right)/c\right]$ and $E\left[{\left(\hat{c}-c\right)}^{2}\right]$, respectively. (*c* denotes the true cut-point and $\hat{c}$ denotes the estimated cut-point by the method.)

In order to estimate the standard deviation and the confidence interval (CI) for the optimal cut-point, the bootstrap resampling technique is applied [14]. To calculate the bootstrap estimate ${\hat{c}}_{B}$, random sampling with replacement is used to draw 200 bootstrap samples within each of the 1000 generated samples. Moreover, to construct a 95% CI for the optimal cut-point, the basic percentile method is applied by taking the 2.5 and 97.5 percentiles of the ${\hat{c}}_{B}$ bootstrap distribution.

The bootstrap estimator of the standard deviation (SD_*B*_) for the estimated cut-point is calculated by taking the standard deviation of the 200 cut-point estimates. Within each of the simulation scenarios, the CIs are subsequently evaluated by computing coverage probability and mean length.

All simulations are done by using R program with the version of 3.2.0. To determine the estimates for Youden index and the point closest-to-(0,1) corner, the pROC library is used [15]. For defining the estimates of the rest of the methods, an R code is written by the author and it can be available upon request.

### 3.1. Simulation Results

Table 3 shows the results for the balanced design under normal homoscedastic distributions. The relative bias values of the previously proposed methods are similar to the results of Rota and Antolini's work [11] except the relative bias of Youden index. In particular for poor classification accuracy scenarios (i.e., *c*_opt_ = 0.25 and 0.52), Youden index has worse performance in the estimation of the optimal cut-point than their results. However, this discrepancy is not seen in the comparison of MSEs. That is, the MSEs of all methods are similar to that of Rota and Antolini's work [11].

When comparing the relative bias and MSE values of the IU method with that of the other methods, it can be easily seen that the IU method has mostly similar performance with the point closest-to-(0,1) corner method and has better performance than the other methods (i.e., lower relative bias and lower MSE values).

For the balanced design under normal homoscedastic distributions, the bootstrap standard deviation, coverage probability, and mean length of the 95% bootstrap CI for the cut-point are shown in Table 4. As in Table 3, the results given in Table 4 are similar to that of Rota and Antolini's work [11]. That is, the SD_*B*_ of the minimum *P* value approach is still greater with respect to that of the other methods and the better classification accuracies provide the narrower 95% bootstrap CIs. The IU method achieves the smallest SD_*B*_ value and the narrowest CIs in most of the scenarios. The coverage probabilities are close to the nominal level for all methods.

The relative bias and MSE results for the unbalanced design under normal homoscedastic distributions are shown in Table 5. Since the true cut-point for the minimum *P* value approach depends on the prevalence of the disease in the sample, different optimal cut-points are used for the comparisons [11]. The relative bias values of all methods are similar to those of Rota and Antolini's work [11], except for the minimum *P* value approach in the lowest classification accuracy scenario (i.e., *c*_opt_ = 0.25). For this scenario the relative bias for the minimum *P* value approach is larger than the bias given in their work. For poor and poor-moderate classification accuracy (i.e., *c*_opt_ = 0.25 and 0.52), the MSE is the lowest for the IU method, and, for moderate-high and high classification accuracy (i.e., *c*_opt_ = 0.84 and 1.28), both the point closest-to-(0,1) corner method and the IU method get the lowest MSE values.

For the unbalanced design under normal homoscedastic distributions, the bootstrap standard deviation, coverage probability, and mean length of the 95% bootstrap CI for the cut-point are given in Table 6. For this scenario, the lowest SD_*B*_ and mean length of the 95% bootstrap CI values are obtained by the point closest-to-(0, 1) corner method and the IU method. As in the comparison of the relative bias and MSE values of the methods (Table 5), for poor and poor-moderate classification accuracy (i.e., *c*_opt_ = 0.25 and 0.52), the IU method gets the lowest SD_*B*_ and mean length, and, for moderate-high and high classification accuracy (i.e., *c*_opt_ = 0.84 and 1.28), both the point closest-to-(0,1) corner in the ROC plane and the IU method get the lowest values. The coverage probabilities are close to the nominal level for all methods.

As it was shown in Rota and Antolini's work [11], under a gamma distribution assumption with a balanced design, the theoretical true cut-points *c*_min⁡*P*_, *c*_*J*_, *c*_CZ_, and *c*_ER_ are all different. For all classification accuracy scenarios, the theoretical true cut-points for the IU method are obtained based on the idea given in the article of Liu [9] (Figure 2). The relative bias values of all methods are similar to those of Rota and Antolini's work [11]. The MSE gets its lowest value in the point closest-to-(0,1) corner and the IU method for all classification accuracy scenarios (Table 7).

For this design (under gamma distributions), the SD_*B*_ and mean length of 95% CI values for the point closest-to-(0,1) corner method and the IU method are lower than the other investigated approaches (Table 8). The coverage probabilities are close to the nominal level for all methods.

In all simulation scenarios, the IU method shows a better performance in the estimation of the optimal cut-point with respect to the other methods. The bootstrap standard deviation and mean length of the 95% bootstrap CI values for the IU method are also minimum among all methods. Thus, for all simulation scenarios, although, in gamma scenarios, the methods do not lead to a common cut-point, in order to identify the optimal cut-point, the IU method is a better alternative than the previous proposed methods.

### 3.2. Cross-Validation of the Optimal Cut-Point

In order to evaluate the significance of the optimally selected cut-point, twofold cross-validation process [16] is used. The procedure is as follows:

1. Generating data with the same properties given in this manuscript
2. Applying all methods to the data and estimating cut-points for all methods
3. Splitting data into two equal subsets, that is, subset I and subset II
4. Applying all methods to subset I and estimating cut-points for all methods
5. Assigning each observation in subset II to either one of two groups by using the cut-point obtained in the previous step
6. Applying all methods to new subset II and estimating cut-points for all methods
7. Assigning each observation in subset I to either one of two groups by using the cut-point obtained in the previous step
8. Applying all methods to the combination of these two subsets and estimating cut-points for all methods
9. Taking the difference between the cut-points obtained at the second step and at the last step

This procedure is applied for 4 scenarios (2 normal and 2 gamma scenarios with the sample size *n*_0_ = *n*_1_ = 50) given in the manuscript. The results are shown in Figure  1 in Supplementary Material available online at https://doi.org/10.1155/2017/3762651. According to the results, for each method, the difference between the optimal cut-points estimated before and after cross-validation is around 0 and the IU method gets the smallest mean absolute difference in all four scenarios.

## 4. Application

A real data obtained from a study in cardiology is used as an example. Yildiran et al. [12] investigated an association between pulse pressure and 2-year cardiovascular death in an entire heart-failure population. They prospectively enrolled 225 (188 male, 37 female) heart-failure patients with NYHA functional classes I–IV, mean age 56.5 [12].

They recorded detailed histories of the 225 patients, including demographic characteristics, cardiovascular (CV) risk factors, and medication usage. The patients were divided into 4 NYHA classes in accordance with their medical histories and the findings upon physical examination and then into 2 groups according to their NYHA class (mild heart failure [classes I-II] and advanced heart failure [classes III-IV]). Levels of serum lipids, glucose, high-sensitivity C-reactive protein, blood urea nitrogen, creatinine, sodium, and potassium were measured by routine laboratory methods. Blood pressures were measured by sphygmomanometer in accordance with published guidelines. Pulse pressure was calculated as the difference between systolic and diastolic blood pressure, and the patients were divided accordingly into 4 quartiles (PP of <35, 35–45, 46–55, or >55 mmHg) [12].

They used ROC analysis to define the cut-point values for pulse pressure, LVEF, plasma sodium value, and heart rate in predicting CV death. In this analysis, 170 patients who had all four measurements at the same time (55 patients' measurements were missing) were included. To get optimal cut-point values, they used ER approach [12].

Supplementary Web-Only Table 1 reports some descriptive statistics of these four measurements. Pulse pressure, LVEF, and plasma sodium levels are significantly lower in dead patients (*n*_1_ = 43) than in alive patients (*n*_0_ = 117) and heart rate is significantly higher in dead patients than in alive patients. According to the results of the Shapiro-Wilk nonparametric normal distribution test, heart rate and plasma sodium are both normally distributed in both groups, LVEF is normally distributed in dead patients and is not normally distributed in alive patients, and pulse pressure is not normally distributed in both groups. For nonnormal distributed variables, the distribution of LVEF in alive patients is left-skewed and the distributions of pulse pressure in both groups are right-skewed. Since the numbers of patients in two groups are not close enough, the design is unbalanced and the ratio between the numbers of patients is similar to the 50 : 100 scenario in the simulation protocol.

**Figure 3:**
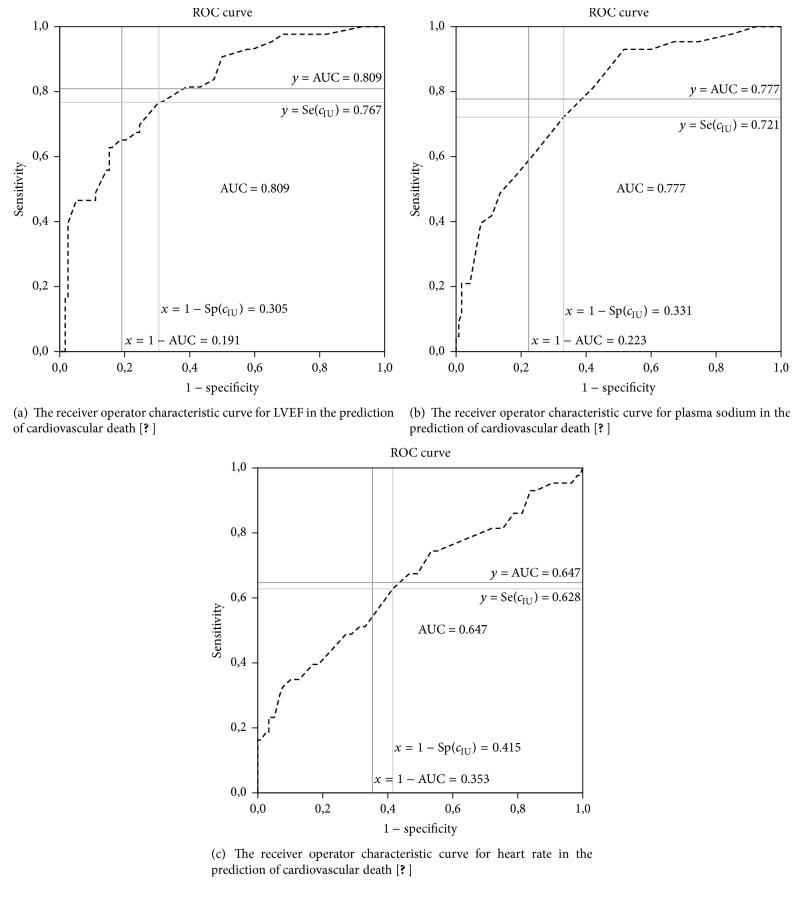
The receiver operator characteristic curves for LVEF, plasma sodium, and heart rate in the prediction of cardiovascular death [12].

In this study, the data obtained from the study by Yildiran et al. [12] is used and all the methods including the IU method are applied to this data. The corresponding results are given in Table 9. The upper part of Table 9 shows the cut-points obtained by using the previously proposed methods. To define the cut-point with the IU method, some of cut-points with their sensitivity and specificity values and AUC value are given. According to this table, the IU method gives the same cut-points with the ER method for different AUC values (Figure 3).

## 5. Conclusions

Defining the optimal cut-point is very important when a continuous variable is considered as a diagnostic marker. Getting optimal classification level depends on the point chosen for diagnosis. The criteria for optimality can change according to the aim of the study. However, as a general rule, minimizing the total misclassification rates is a good approach. With IU method, since the difference between sensitivity and specificity values is minimum, this condition is met most of the time.

According to the results given in the tables, the proposed IU method can be a better alternative for defining the cut-point. When the definition of optimal point is stated as the point that minimizes the misclassification rates or the point that equalizes the values of sensitivity and specificity, the IU method is better than the other methods in most of the comparison scenarios. This conclusion does not change with the distribution of biomarker or the homogeneity of variances of biomarkers. The changes in the sample size and the AUC values may affect but not alter the interpretation.

The IU method uses the absolute difference between diagnostic accuracy measures and AUC value instead of using the Euclidean distance. The reason behind this idea is to provide the simplicity in defining the point as optimal. With the IU method, one can easily identify the optimal cut-point only by checking whether the sensitivity and specificity values are close enough to AUC value or not. That is, the complex calculations are not necessary for the IU method.

When the relative bias and MSE values of the IU method are compared with the previous methods, it is seen that the IU method is better than the others. Thus this method can be used for defining the optimal cut-point value especially when the sample sizes of the two groups are equal and the AUC value is high. (i.e., higher than 0.7).

A common practice is to select a cut-point which defines two risk groups for a continuously measured biomarker [16]. A cut-point for a biomarker is meaningful for the clinicians when it is clinically interpretable and understandable. Clinical meaning for a cut-point can be explained by using its accuracy, that is, true classification rate. Among all the methods, only two of them, the Youden index and the concordance probability, are based on the maximization of this rate. Thus, these methods provide interpretable cut-points.

The point closest to (0,1) point on the ROC curve method involves a quadratic term and clinical meaning of this term is unknown. Despite the lack of clinical meaning, it is shown in the literature that this method is superior to the other methods in estimating the true cut-point [11].

The IU method, like the Youden index and the concordance probability, tries to minimize the misclassification rate. Hence, it also provides an interpretable cut-point. In this study, it is shown that the IU method performs better than (or equal to) the point closest to (0,1) point on the ROC curve method. Therefore, the use of the IU method is advised to get more interpretable and better optimized cut-point.

The IU method provides a cut-point whose sensitivity and specificity are equally high. This means that, in a cut-point determination process, if sensitivity and specificity are valued equally, the IU method seems to be the best option among all other methods.

## Supplementary Material

Supplementary Table 1: Descriptive statistics of the application example of cut-point finding for Pulse pressure, LVEF, Plasma sodium level and Heart rate in prediction of mortality, from Yildiran et al. (2010).
Supplementary Figure 1: The difference between the optimal cut-points estimated before and after cross-validation is around 0 and the IU method gets the smallest mean absolute difference in all four scenarios.

## Figures and Tables

**Table 1:** 

	*X* ≤ *c*	*X* > *c*
*E* = 0	*s*	*r*
*E* = 1	*u*	*v*

**Table 2:** Some of the cut-points with their sensitivity and specificity values obtained from artificial data.

Cut-point	Specificity	Sensitivity
⋯	⋯	⋯
3.095	0.44	0.92
2.986	0.48	0.92
2.727	0.52	0.92
2.527	0.56	0.92
2.478	0.60	0.92
2.416	0.64	0.92
2.331	0.68	0.92
2.284	0.72	0.92
2.262	0.76	0.92
2.243	0.80	0.92
2.191	0.84	0.92
2.079	0.88	0.92
1.985	0.92	0.92
1.944	0.92	0.88
1.897	0.92	0.84
1.836	0.92	0.80
1.741	0.92	0.76
⋯	⋯	⋯

**Table 3:** Relative bias and mean square error (MSE) of all methods. The normal homoscedastic balanced scenario^a^.

*c*_opt_	Sample sizes	Minimum *P* value		Youden index		Concordance probability		Point closest-to-(0-1) corner		Index of Union	
	*n*_1_ = *n*_2_	Relative bias	MSE	Relative bias	MSE	Relative bias	MSE	Relative bias	MSE	Relative bias	MSE
0. 25	50	0.0080	0.5622	0.3088	0.2358	0.0432	0.0696	0.0357	0.0513	0.0306	0.0191
	100	0.1303	0.4604	0.3129	0.1675	0.0588	0.0428	0.0526	0.0315	0.0505	0.0116
	200	−0.0174	0.3652	0.1510	0.1158	0.0145	0.0259	0.0221	0.0195	0.0262	0.0074

0.52	50	0.0068	0.2307	0.1161	0.1266	0.0066	0.0676	0.0112	0.0427	0.0172	0.0265
	100	−0.0314	0.1752	0.0732	0.0783	0.0072	0.0392	0.0084	0.0258	0.0035	0.0201
	200	−0.0073	0.1190	0.0438	0.0490	0.0078	0.0242	0.0119	0.0145	0.0109	0.0153

0.84	50	0.0040	0.1263	0.0563	0.0822	−0.0026	0.0557	−0.0038	0.0369	−0.0016	0.0341
	100	0.0140	0.0839	0.0476	0.0538	0.0023	0.0372	0.0024	0.0219	0.0020	0.0268
	200	−0.0036	0.0631	0.0282	0.0362	0.0039	0.0237	0.0029	0.0128	0.0042	0.0214

1.28	50	0.0011	0.0872	0.0292	0.0676	0.0015	0.0563	0.0032	0.0410	0.0033	0.0467
	100	0.0018	0.0558	0.0269	0.0444	0.0025	0.0368	0.0029	0.0245	0.0030	0.0336
	200	−0.0028	0.0343	0.0170	0.0248	0.0017	0.0205	0.0013	0.0119	0.0021	0.0200

^a^ *X*_1_ ~ *N*  (*μ*_1_, 1),  *X*_0_ ~ *N*  (0,1), and *μ*_1_ was taken as 0.51, 1.05, 1.68, and 2.56, respectively.

**Table 4:** Bootstrap standard deviation, coverage probability, and mean length of the 95% confidence interval estimation of all methods. The normal homoscedastic balanced scenario^a^.

*c*_opt_	Sample sizes	Minimum *P* value			Youden index			Concordance probability			Point closest-to-(0-1) corner			Index of Union		
	*n*_1_ = *n*_2_	SD_*B*_	Coverage	Mean length	SD_*B*_	Coverage	Mean length	SD_*B*_	Coverage	Mean length	SD_*B*_	Coverage	Mean length	SD_*B*_	Coverage	Mean length
0.25	50	0.7473	0.964	2.7559	0.4776	0.969	1.8484	0.2633	0.971	1.0333	0.2262	0.969	0.8837	0.1380	0.974	0.5502
	100	0.6767	0.967	2.5637	0.4017	0.968	1.5553	0.2061	0.973	0.8074	0.1767	0.972	0.7019	0.1070	0.966	0.4173
	200	0.6039	0.968	2.3059	0.338	0.969	1.3063	0.1606	0.959	0.5896	0.1393	0.967	0.5359	0.0858	0.970	0.3325

0.52	50	0.4811	0.968	1.8521	0.3507	0.969	1.3411	0.2602	0.971	1.0181	0.2071	0.973	0.8187	0.1630	0.973	0.6476
	100	0.4186	0.968	1.5878	0.2778	0.972	1.1006	0.1982	0.969	0.7566	0.1607	0.970	0.6233	0.1420	0.969	0.5489
	200	0.3434	0.970	1.3399	0.219	0.975	0.8786	0.1551	0.973	0.6139	0.1199	0.971	0.4717	0.1231	0.965	0.4623

0.84	50	0.3556	0.969	1.3557	0.2826	0.969	1.0837	0.2354	0.973	0.9261	0.1921	0.974	0.7692	0.1845	0.976	0.7475
	100	0.2899	0.970	1.1106	0.2289	0.972	0.8922	0.1930	0.972	0.7595	0.1477	0.972	0.5843	0.1637	0.970	0.6491
	200	0.2515	0.970	0.9619	0.1890	0.972	0.7375	0.1538	0.975	0.6154	0.1132	0.971	0.4356	0.1465	0.970	0.5601

1.28	50	0.2958	0.965	1.1106	0.2578	0.974	1.0262	0.2379	0.974	0.9419	0.2027	0.973	0.8198	0.2166	0.974	0.8668
	100	0.2359	0.968	0.8973	0.2074	0.972	0.8112	0.1916	0.975	0.7679	0.1566	0.971	0.6157	0.1831	0.973	0.7294
	200	0.1851	0.971	0.7240	0.1556	0.970	0.6141	0.1431	0.970	0.5683	0.1094	0.972	0.4272	0.1414	0.969	0.5607

^a^ *X*_1_ ~*N*  (*μ*_1_, 1), *X*_0_~*N*  (0,1), and *μ*_1_ was taken as 0.51, 1.05, 1.68, and 2.56, respectively.

**Table 5:** Relative bias and mean square error (MSE) of all methods. The normal homoscedastic unbalanced scenario^a^.

*c*_opt_	*c*_min⁡*P*_	Sample sizes		Minimum *P* value		Youden index		Concordance probability		Point closest-to-(0-1) corner		Index of Union	
		*n*_1_	*n*_2_	Relative bias	MSE	Relative bias	MSE	Relative bias	MSE	Relative bias	MSE	Relative bias	MSE
0.25	0.39	50	100	0.1750	0.5041	0.2046	0.1813	0.1042	0.0520	0.1007	0.0341	0.0686	0.0075
	0.46	50	150	0.2986	0.5124	0.1683	0.1708	0.1142	0.0515	0.1324	0.0338	0.0923	0.0079
	0.51	50	200	0.2955	0.5517	0.1420	0.1741	0.1483	0.0484	0.1544	0.0320	0.1059	0.0074

0.52	0.75	50	100	0.0361	0.1963	0.1061	0.0921	0.0572	0.0469	0.0481	0.0256	0.0535	0.0161
	0.87	50	150	0.0613	0.1922	0.1141	0.0904	0.0588	0.0435	0.0746	0.0255	0.0597	0.0162
	0.96	50	200	0.0601	0.2060	0.0926	0.0785	0.0690	0.0399	0.0778	0.0233	0.0696	0.0159

0.84	1.09	50	100	0.0006	0.0952	0.0411	0.0597	0.0157	0.0414	0.0219	0.0219	0.0162	0.0255
	1.23	50	150	0.0030	0.0956	0.0498	0.0580	0.0313	0.0423	0.0375	0.0232	0.0328	0.0259
	1.33	50	200	0.0028	0.0996	0.0440	0.0536	0.0345	0.0374	0.0409	0.0201	0.0342	0.0248

1.28	1.50	50	100	−0.0123	0.0581	0.0350	0.0460	0.0177	0.0390	0.0225	0.0254	0.0195	0.0325
	1.63	50	150	−0.0055	0.0528	0.0378	0.0479	0.0254	0.0414	0.0298	0.0242	0.0270	0.0342
	1.72	50	200	0.0007	0.0507	0.0469	0.0477	0.0380	0.0422	0.0386	0.0257	0.0368	0.0356

^a^ *X*_1_ ~*N*  (*μ*_1_, 1), *X*_0_~*N*  (0,1), and *μ*_1_ was taken as 0.51, 1.05, 1.68, and 2.56, respectively.

**Table 6:** Bootstrap standard deviation, coverage probability, and mean length of the 95% confidence interval estimation of all methods. The normal homoscedastic unbalanced scenario^a^.

*c*_opt_	*c*_min⁡*P*_	Sample sizes		Minimum *P* value			Youden index			Concordance probability			Point closest-to-(0-1) corner			Index of Union		
		*n*_1_	*n*_2_	SD_B_	Coverage	Mean length	SD_*B*_	Coverage	Mean length	SD_*B*_	Coverage	Mean length	SD_*B*_	Coverage	Mean length	SD_*B*_	Coverage	Mean length
0.25	0.39	50	100	0.6968	0.968	2.6365	0.4205	0.969	1.6059	0.2296	0.968	0.8881	0.1851	0.971	0.7191	0.0874	0.967	0.3504
	0.46	50	150	0.7027	0.962	2.6012	0.4108	0.969	1.6012	0.2247	0.967	0.8751	0.1804	0.967	0.6892	0.0856	0.964	0.3336
	0.51	50	200	0.7277	0.960	2.6871	0.4157	0.972	1.6151	0.2171	0.967	0.8514	0.1747	0.964	0.6633	0.0821	0.959	0.3174

0.52	0.75	50	100	0.4419	0.971	1.7011	0.2983	0.969	1.1461	0.2151	0.969	0.8252	0.1576	0.967	0.6068	0.1234	0.967	0.4666
	0.87	50	150	0.4340	0.971	1.6816	0.2943	0.966	1.1459	0.2061	0.971	0.8003	0.1548	0.975	0.6226	0.1231	0.961	0.4671
	0.96	50	200	0.4504	0.968	1.7346	0.2757	0.966	1.0601	0.1964	0.971	0.7882	0.1470	0.967	0.5685	0.1207	0.968	0.4561

0.84	1.09	50	100	0.3081	0.970	1.1866	0.2416	0.971	0.9343	0.2031	0.971	0.8028	0.1472	0.970	0.5754	0.1594	0.967	0.6031
	1.23	50	150	0.3094	0.971	1.2033	0.2375	0.970	0.9123	0.2044	0.973	0.8064	0.1489	0.971	0.5805	0.1585	0.969	0.6001
	1.33	50	200	0.3151	0.969	1.2265	0.2292	0.972	0.8917	0.1914	0.972	0.7639	0.1379	0.968	0.5261	0.1547	0.964	0.5648

1.28	1.50	50	100	0.2408	0.968	0.9199	0.2092	0.972	0.8294	0.1955	0.974	0.7846	0.1563	0.971	0.6093	0.1780	0.972	0.6941
	1.63	50	150	0.2298	0.973	0.9089	0.2131	0.972	0.8423	0.2004	0.972	0.7983	0.1506	0.973	0.5962	0.1818	0.970	0.7011
	1.72	50	200	0.2254	0.968	0.8716	0.2101	0.966	0.8096	0.1998	0.964	0.7671	0.1526	0.970	0.5929	0.1825	0.965	0.6890

^a^ *X*_1_ ~*N*  (*μ*_1_, 1),  *X*_0_~*N*  (0,1), and *μ*_1_ was taken as 0.51, 1.05, 1.68, and 2.56, respectively.

**Table 7:** Relative bias and mean square error (MSE) of all methods. The gamma balanced scenario^a^.

*c*_min⁡*P*_	*c*_*J*_	*c*_CZ_	*c*_ER_	*c*_IU_	Sample sizes	Minimum *P* value		Youden index		Concordance probability		Point closest-to-(0-1) corner		Index of Union	
					*n*_1_ = *n*_2_	Relative bias	MSE	Relative bias	MSE	Relative bias	MSE	Relative bias	MSE	Relative bias	MSE
0.80	1.12	1.35	1.38	1.42	50	0.4290	0.5491	0.0862	0.2095	0.0174	0.0713	0.0100	0.0521	0.0243	0.0133
					100	0.2735	0.3001	0.0565	0.1321	0.0126	0.0464	0.0016	0.0314	0.0195	0.0078
					200	0.1934	0.1813	0.0395	0.0885	0.0116	0.0305	0.0024	0.0211	0.0156	0.0065

1.73	1.79	1.81	1.82	1.78	50	0.0735	0.4727	0.0269	0.2260	0.0168	0.1108	0.0160	0.0648	0.0365	0.0401
					100	0.0454	0.3347	0.0229	0.1328	0.0099	0.0655	0.0126	0.0385	0.0272	0.0303
					200	0.0361	0.2276	0.0248	0.0932	0.0034	0.0439	0.0084	0.0248	0.0249	0.0282

2.54	2.45	2.41	2.36	2.41	50	0.0099	0.4109	−0.0262	0.2420	0.0064	0.1607	0.0099	0.0919	−0.0087	0.0840
					100	−0.0073	0.2771	−0.0245	0.1554	−0.0108	0.1103	−0.0006	0.0553	−0.0100	0.0699
					200	0.0042	0.1955	−0.0170	0.1107	−0.0094	0.0695	−0.0037	0.0343	0.0026	0.0600

3.51	3.42	3.38	3.24	3.30	50	−0.0206	0.4773	−0.0148	0.3108	−0.0061	0.2591	0.0171	0.1828	−0.0091	0.1859
					100	−0.0157	0.3061	−0.0066	0.2221	0.0004	0.1957	0.0127	0.1112	0.0064	0.1561
					200	−0.0214	0.2101	−0.0028	0.1463	0.0004	0.1291	0.0095	0.0599	0.0148	0.1107

^a^ *X*_1_ ~*G*  (2.5, *β*_1_), *X*_0_~*G*  (1.5,1), and *β*_1_ was taken as 0.79, 1.22, 1.97, and 3.82, respectively; for the true cut-points *c*_min⁡*P*_, *c*_*J*_, *c*_CZ_, and *c*_ER_, the results of Rota and Antolini's were used; for the true cut-point *c*_IU_, the objective function is maximized.

**Table 8:** Bootstrap standard deviation, coverage probability, and mean length of the 95% confidence interval estimation of all methods. The gamma balanced scenario^a^.

*c*_min⁡*P*_	*c*_*J*_	*c*_CZ_	*c*_ER_	*c*_IU_	Sample sizes	Minimum *P* value			Youden index			Concordance probability			Point closest-to-(0-1) corner			Index of Union		
					*n*_1_ = *n*_2_	SD_*B*_	Coverage	Mean length	SD_*B*_	Coverage	Mean length	SD_*B*_	Coverage	Mean length	SD_*B*_	Coverage	Mean length	SD_*B*_	Coverage	Mean length
0.80	1.12	1.35	1.38	1.41	50	0.6613	0.878	2.5752	0.4468	0.934	1.7152	0.2661	0.969	1.0299	0.2284	0.966	0.8804	0.1105	0.971	0.4336
					100	0.5048	0.893	1.9022	0.3585	0.943	1.3967	0.2142	0.968	0.8394	0.1771	0.964	0.6805	0.0838	0.970	0.3202
					200	0.3997	0.918	1.5638	0.2952	0.946	1.1355	0.1737	0.969	0.6829	0.1450	0.968	0.5751	0.0774	0.960	0.2872

1.73	1.79	1.81	1.82	1.74	50	0.6767	0.934	2.5512	0.4719	0.950	1.8199	0.3317	0.964	1.3298	0.2529	0.966	0.9481	0.1894	0.971	0.7289
					100	0.5719	0.942	2.2325	0.3617	0.956	1.4270	0.2551	0.968	0.9812	0.1946	0.965	0.7422	0.1674	0.961	0.6163
					200	0.4730	0.958	1.8935	0.3026	0.959	1.1626	0.2096	0.965	0.8076	0.1564	0.958	0.5983	0.1618	0.950	0.5822

2.54	2.45	2.41	2.36	2.48	50	0.6409	0.966	2.4846	0.4866	0.970	1.9271	0.4002	0.959	1.5788	0.3024	0.968	1.1684	0.2891	0.971	1.1234
					100	0.5257	0.958	1.9721	0.3901	0.967	1.5215	0.3310	0.964	1.2616	0.2347	0.968	0.8941	0.2631	0.969	0.9996
					200	0.4422	0.965	1.6817	0.3296	0.964	1.3089	0.2624	0.967	1.0213	0.1849	0.968	0.7279	0.2452	0.970	0.9433

3.51	3.42	3.38	3.24	3.37	50	0.6881	0.964	2.6282	0.5559	0.963	2.2071	0.5091	0.959	1.9967	0.4241	0.957	1.6429	0.4295	0.964	1.6911
					100	0.5491	0.968	2.0911	0.4706	0.962	1.8332	0.4421	0.963	1.7384	0.3315	0.970	1.2972	0.3947	0.969	1.5351
					200	0.4511	0.968	1.7641	0.3823	0.957	1.5002	0.3594	0.958	1.4143	0.2427	0.966	0.9504	0.3292	0.965	1.2568

^a^ *X*_1_ ~*G*  (2.5, *β*_1_),  *X*_0_~*G*  (1.5,1), and *β*_1_ was taken as 0.79, 1.22, 1.97, and 3.82, respectively; for the true cut-points *c*_min⁡*P*_, *c*_*J*_, *c*_CZ_, and *c*_ER_, the results of Rota and Antolini's were used; for the true cut-point *c*_IU_, the empirically estimated objective function is maximized.

**Table 9:** The true cut-point estimates obtained by all the methods: some of cut-points and the AUC values for pulse pressure, LVEF, plasma sodium level and heart rate in prediction of mortality.

	Pulse pressure	LVEF	Plasma sodium	Heart rate
	Point (Se, Sp)	Point (Se, Sp)	Point (Se, Sp)	Point (Se, Sp)
Youden index	30 (83.7, 79.7)	0.264 (62.8, 84.7)	137 (93.0, 48.3)	99 (32.6, 91.5)
ER	30 (83.7, 79.7)	0.295 (76.7, 69.5)	135 (72.1, 66.9)	85 (62.8, 58.5)
Min *P* value	24 (98.3, 53.5)	0.235 (46.5, 94.9)	130 (39.5, 92.4)	115 (16.3, 99.2)
CZ	30 (83.7, 79.7)	0.295 (76.7, 69.5)	135 (72.1, 66.9)	85 (62.8, 58.5)

Some cut-off points with their sensitivity and specificity values	⋯ 24 (53.5, 98.3) 27 (81.4, 79.7) 30 (83.7, 79.7) 34 (83.7, 77.1) 37 (100, 39.0) …	⋯ 0.272 (65.1, 81.4) 0.282 (67.4, 76.3) 0.290 (69.8, 75.4) 0.295 (76.7, 69.5) 0.303 (81.4, 61.0) ⋯	⋯ 133 (53.5, 82.2) 134 (60.5, 76.3) 135 (72.1, 66.9) 136 (81.4, 57.6) 137 (93.0, 48.3) ⋯	⋯ 84 (67.4, 53.4) 85 (62.8, 58.5) 86 (58.1, 61.9) 87 (51.2, 68.6) ⋯

Index of Union	30 (83.7, 79.7)	0.295 (76.7, 69.5)	135 (72.1, 66.9)	85 (62.8, 58.5)

AUC	0.892	0.809	0.777	0.647

*Note.* Point: cut-point; Se: sensitivity; Sp: specificity; AUC: the area under the curve.

## Appendix

### Identification of the True Theoretical Cut-Point for the IU Method under the Normal Homoscedastic Distribution Case

Let us consider the normal homoscedastic distribution scenario, where *X*_*D*_ ~ *N*  (*μ*_*D*_, *σ*_*D*_),  *D* = 0, 1 (assuming *μ*_1_ > *μ*_0_ = 0 and *σ*_0_ = *σ*_1_ = 1). Then, the conditional distribution of the quantitative variable *X* in group *D* is *F*_*D*_(*c*) = *P*(*X* ≤ *c*∣*D*) for *D* = 0, 1.

In particular, at cut-point *c*, specificity Sp(*c*) = *F*_0_(*c*), and sensitivity Se(*c*) = 1 − *F*_1_(*c*). Then the IU function can be written as one of the following forms (according to the difference in the absolute value):

- IU(*c*) = *F*_0_(*c*) − *F*_1_(*c*) + 1 − 2*∗*AUC
- IU(*c*) = 1 − *F*_0_(*c*) − *F*_1_(*c*)
- IU(*c*) = *F*_0_(*c*) + *F*_1_(*c*) − 1
- IU(*c*) = 2*∗*AUC − 1 − *F*_0_(*c*) + *F*_1_(*c*)

That is, IU(*c*) = *αF*_0_(*c*) + *βF*_1_(*c*) + *γ* where *α*, *β* and *γ* are arbitrary (*α*, *β* = −1 or 1,   −1 ≤ *γ* ≤ 1). Thus this formulation is general form of the Youden Index. So, the cut-point which optimizes the IU function can be obtained by taking the first derivative of IU(*c*), ∂IU(*c*)/∂*c* = *αf*_0_(*c*) + *βf*_1_(*c*), where *f*_*D*_(*c*) = ∂*F*_*D*_(*c*)/∂*c* are the normal probability density functions for diseased and nondiseased subjects. Since the normal distribution is symmetric, *f*_0_ = −*f*_0_ for the standard normal distribution and thus the root of ∂IU(*c*)/∂*c* = 0 is *c*_IU_ = *μ*_1_/2.
