# Supplementary material for: Defining an Optimal Cut-Point Value in ROC Analysis: An Alternative Approach
Source: Comput Math Methods Med. 2017 May 31;2017:3762651. doi: 10.1155/2017/3762651 (PMC5470053; doi:10.1155/2017/3762651)
Supplement: Supplementary file 1 — Supplementary Table 1: Descriptive statistics of the application example of cut-point finding for Pulse pressure, LVEF, Plasma sodium level and Heart rate in prediction of mortality, from Yildiran et al. (2010). Supplementary Figure 1: The difference between the optimal cut-points estimated before and after cross-validation is around 0 and the IU method gets the smallest mean absolute difference in all four scenarios. [file 3762651.f1.docx]

**Supplementary web only Table 1**. Descriptive statistics of the application example of cut-point finding for Pulse pressure, LVEF, Plasma sodium level and Heart rate in prediction of mortality, from Yildiran et al. (2010).

| Variable | Alive^a^  (n=117) | Exitus^a^  (n=43) | p^b^ | p^c^ | p^d^ | p^e^ |
| --- | --- | --- | --- | --- | --- | --- |
| Pulse Pressure (mmHg) | 44.0±12.2 | 27.4±7.0 | <0.001 | <0.001 | <0.001 | 0.008 |
| Heart Rate (beats/min) | 83.4±10.9 | 91.5±16.4 | 0.004 | 0.103 | 0.183 | <0.001 |
| LVEF | 0.325±0.058 | 0.254±0.058 | <0.001 | 0.272 | <0.001 | 0.851 |
| Plasma Sodium (mmol/L) | 137.2±4.26 | 131.8±5.45 | <0.001 | 0.102 | 0.120 | 0.044 |

^a^ Mean and Standard Deviation

^b^ Student T test or Mann Whitney Test for equality of means

^c^ Shapiro-Wilk test for the distribution of the variable of dead patients being normal

^d^ Shapiro-Wilk test for the distribution of the variable alive patients being normal

^e^ Levene’s test for equality of variances

Pulse pressure, LVEF and plasma sodium levels are significantly lower in dead patients (n_1_=43) than in alive patients (n_0_=117) and heart rate is significantly higher in dead patients than in alive patients. According to the results of the Shapiro–Wilk non-parametric normal distribution test, heart rate and plasma sodium are both normally distributed in both two groups, LVEF is normally distributed in dead patients and is not normally distributed in alive patients and pulse pressure is not normally distributed in both two groups. For non-normal distributed variables, the distribution of LVEF in alive patients is left skewed (i.e., exponential) and the distributions of pulse pressure in both two groups are right skewed (i.e., gamma). Since the numbers of patients in both two groups are not close enough, the design is unbalanced and the ratio between the numbers of patients in groups is similar to the 50:100 scenario in the simulation protocol.

**Supplementary web only Figure 1**. The differences between the optimal cut-points estimated before and after cross validation.


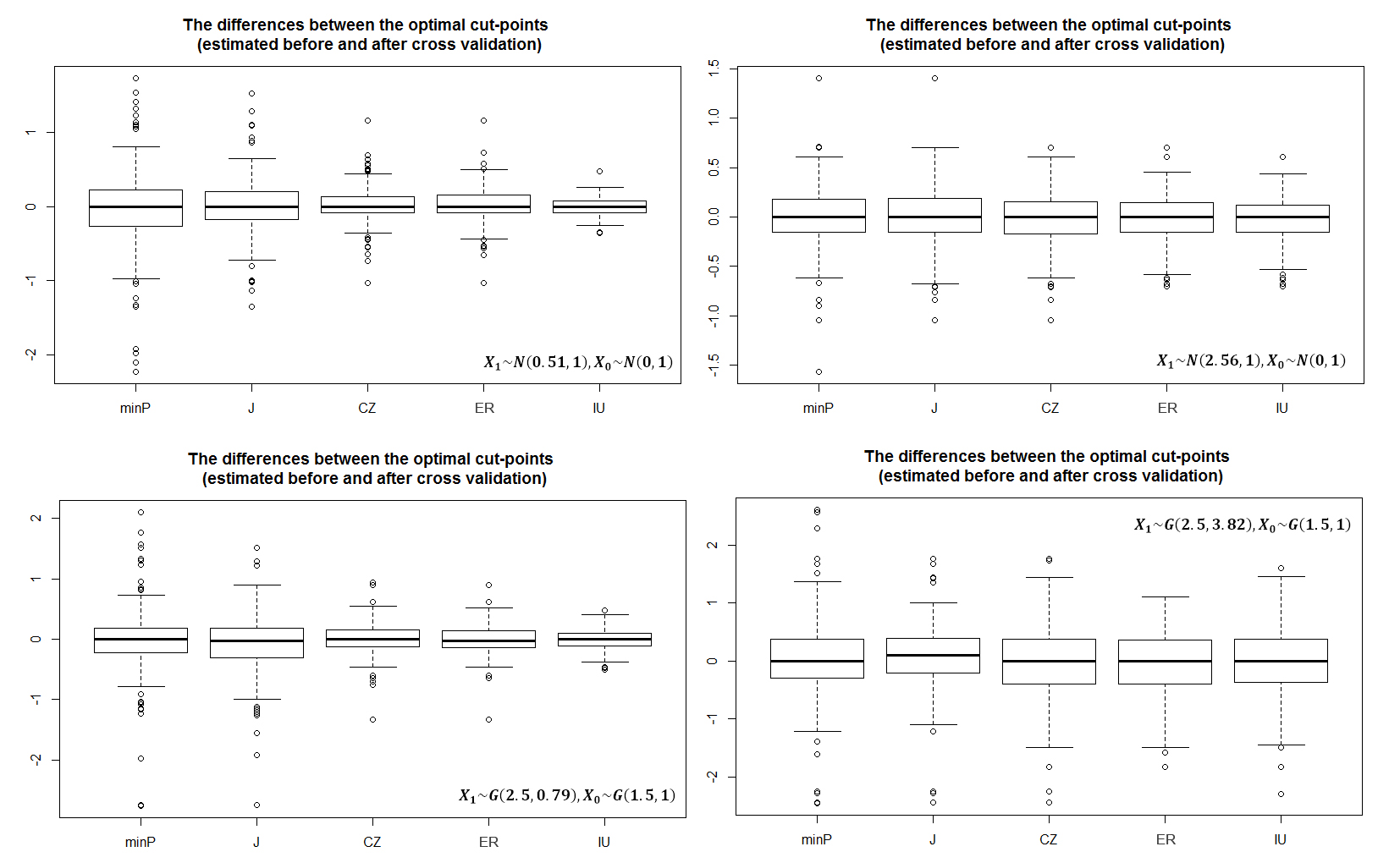


minP: Minimum P-value approach, J: Youden index, CZ: Concordance probability, ER: The closest to (0,1) criteria, IU: Index of Union

The cross-validation procedure serves to assess the significance of the cut-point [16]. For each method, the difference between the optimal cut-points estimated before and after cross validation is around 0. According to the results, the IU method gets the smallest mean absolute difference in all four scenarios.
